# A Plea for the Institution of a New Special Course of Instruction in Veterinary Colleges

**Published:** 1896-06

**Authors:** Samuel Goldstein

**Affiliations:** Attending Surgeon, New York Throat and Nose Hospital, Etc.


### A PLEA FOR THE INSTITUTION OF A NEW SPECIAL COURSE OF INSTRUCTION IN VETERINARY COLLEGES.

By SAMUEL GOLDSTEIN, A.B., M.D.,
ATTENDING SURGEON, NEW YORK THROAT AND NOSE HOSPITAL, ETC.

Steady advancement in scientific work has in recent times characterized the progress made in veterinary medicine and surgery. Pathology, bacteriology, ophthalmology, and other special lines of research have been accorded graded courses in the colleges, and instruction, both didactic and practical, has been instituted and pursued in a consistently thorough manner. With this progressive spirit, research, and the practical application of those principles derived therefrom, has gone further and further in the direction of a thorough accomplishment of the end in view. With these bright prospects for progressive veterinary medicine, to what pinnacle of perfection the coming student may be trained only the future can reveal. However, notwithstanding the zeal already displayed in the aforementioned lines, another special course appeals to us in the strongest of terms, and in the very near future will, I feel assured, assume the proper position its importance demands. As a specialty in veterinary medicine, it is undoubtedly but in its infancy; and, therefore, the time is not yet ripe for the full demonstration of its practical importance.

The diseases which assail the air-passages, directly and indirectly, are many and various; and we can safely state that the lower air-passages have undoubtedly received the benefit of the attention they properly deserve. But, may I ask, what attitude has been assumed in the consideration of the upper air-passages? As far as I have been able to ascertain, the barest mention has been made in the text-books and reports upon veterinary medicine and surgery. I feel fully convinced that it is no idle assertion that many of the diseases which assail the lower air-passages have had their nidus, or are at least to a great extent, dependent upon some local disease of the nose, pharynx, or larynx.

With the evidence of these bare facts, can we at the present day turn aside or overlook so important a respiratory channel as the nose and throat?

Comparative medicine and surgery have produced results at one time undreamed of. To-day physiological effects of drugs, new operative procedures, etc., have been experimented with upon animals, and the results derived therefrom employed for the benefit of mankind. Might not a reversed application of known principles in rhinology and laryngology achieved upon man be employed in the study and application upon quadrupeds, at least upon our domestic animals? Cases of common distemper occurring daily in animals would not be so accepted in human medical circles. The acute rhinitis, as well as the general condition, would receive the attention of the rhinologist.

Why should we not apply similar principles in the treatment of the animal? Epistaxis, to be properly treated, would have its origin carefully sought, and whenever passive, when found due to a local lesion, that particular pathological condition would receive prompt attention, both to treatment of the emergency and in the endeavor to prevent its possible recurrence. If due to the presence of polypi or septal ulceration, should not these local pathological conditions receive appropriate local treatment? Septal deformities, nasal and laryngeal neoplasms, and catarrhal conditions of the nose, pharynx, and larynx, should be recognized by inspection, and receive prompt and, whenever possible, early treatment. But how can this end be accomplished? is the natural query.

In order to recognize these lesions they must be sought for by proper means. Rhinoscopic, both anterior and posterior, as well as laryngoscopic examinations must be thoroughly made. I do not believe that in the curriculum of the colleges of veterinary medicine any special course is given upon diseases of the upper air-passages, notwithstanding its almost universally accepted importance. The time has arrived, however, when every properly equipped physician of any school of medicine must be prepared to meet cases involving the nose and throat, whether for local conditions only or in connection with systemic lesions, and be able to make scientific examinations of these parts.

The only solution to this problem, I believe, lies in the hands of the colleges. A special course of didactic and practical instruction in this special line, and, furthermore, provision for post-graduate instruction, will within the near future become incumbent upon every well-regulated institution of medical training.

